# Impacts of Subchronic and Mild Social Defeat Stress on Plasma Putrefactive Metabolites and Cardiovascular Structure in Male Mice

**DOI:** 10.3390/ijms24021237

**Published:** 2023-01-08

**Authors:** Atsushi Toyoda, Kina Kawakami, Yuto Amano, Hideaki Nishizawa, Shin-ichi Nakamura, Takahiro Kawase, Yuta Yoshida, Hodaka Suzuki, Takamitsu Tsukahara

**Affiliations:** 1 Department of Food and Life Sciences, College of Agriculture, Ibaraki University, Ami 300-0393, Japan; 2 United Graduate School of Agricultural Science, Tokyo University of Agriculture and Technology, Fuchu 183-8509, Japan; 3 Laboratory of Veterinary Pathology, Faculty of Veterinary Medicine, Okayama University of Science, Ehime 794-8555, Japan; 4 Kyoto Institute of Nutrition & Pathology, Ujitawara, Kyoto 610-0231, Japan

**Keywords:** fibrosis, heart, mouse, putrefactive metabolite, social defeat, stress

## Abstract

Psychosocial stress precipitates mental illnesses, such as depression, and increases the risk of other health problems, including cardiovascular diseases. In this study, we observed the effects of psychosocial stress on the histopathological features of systemic organs and tissues in a mouse psychosocial stress model, namely the subchronic and mild social defeat stress (sCSDS) model. There were several pathological findings in the tissues of both sCSDS and control mice. Mild fibrosis of the heart was observed in sCSDS mice but not in control mice. Extramedullary hematopoiesis in the spleen and hemorrhage in the lungs were observed in both the control and sCSDS mice. Focal necrosis of the liver was seen only in control mice. Furthermore, putrefactive substances in the blood plasma were analyzed because these metabolites originating from intestinal fermentation might be linked to heart fibrosis. Among them, plasma *p*-cresyl glucuronide and *p*-cresyl sulfate concentrations significantly increased owing to subchronic social defeat stress, which might influence cardiac fibrosis in sCSDS mice. In conclusion, several pathological features such as increased cardiac fibrosis and elevated plasma putrefactive substances were found in sCSDS mice. Thus, sCSDS mice are a potential model for elucidating the pathophysiology of psychosocial stress and heart failure.

## 1. Introduction

Chronic psychosocial stress precipitates mental disorders, including depression, and affects approximately 280 million patients worldwide [1]. There is also evidence of an association between depression and peripheral diseases, such as cardiac and cerebrovascular diseases [2,3,4]. Clinical studies have shown that stress is a risk factor for cardiovascular disease [5,6,7]. Therefore, the impact of psychosocial stress on peripheral tissues and organs, including the cardiovascular system, should be investigated to reduce the risk of stress-induced cardiovascular deficits, such as heart attacks.

Studies on the deleterious impacts of psychosocial stress use various animal stress models; specifically, models where mice are subjected to social defeat stress are well-characterized [8,9,10]. Socially defeated mice show depression-like phenotypes, including social avoidance, anhedonia, nesting deficit, and increased peripheral and central inflammation [9,11,12]. C57BL6 mice are frequently used in the social defeat model, while BALB/c (BALB) mice are also used because of their susceptibility to stress [13,14]. The central nervous system has been well-studied using social defeat models, but there are several pathogeneses in the peripheral tissues and metabolism—including the heart, liver, and intestine—that should be investigated [15,16,17,18,19].

Putrefactive compounds such as indole, skatole, *p*-cresol, phenol, and trimethylamine are produced by the gut microbiota [20,21]. A portion of them are absorbed from the intestine and carried to the liver by the blood circulation. Because these putrefactive compounds are highly toxic, they are conjugated and detoxicated in the liver [22,23]. However, these putrefactive metabolites (e.g., phenyl glucuronide, indoxyl glucuronide, *p*-cresyl glucuronide, phenyl sulfate, indoxyl sulfate, *p*-cresyl sulfate, and trimethylamine *N*-oxide (TMAO)) still have adverse effects on the organism, because these putrefactive metabolites are inflammation factors in the mammalian body [24,25]. Therefore, these putrefactive metabolites are associated with some chronic inflammation diseases, such as chronic kidney disease (CKD) [26,27].

In this study, we attempted to elucidate the pathological features of both the brain and peripheral tissues of male BALB mice subjected to sCSDS. Furthermore, we analyzed blood putrefactive metabolites, including TMAO and *p*-cresyl glucuronide, which might affect the cardiovascular system.

## 2. Results

### 2.1. Feed and Water Intake and Body Weight Gain

Total feed and water intake and body weight gain during the sCSDS period are shown in Table 1. There were no significant differences between the control and sCSDS groups.

### 2.2. Pathological Findings in Tissues

Histopathological analyses of all mice were conducted, and the results are summarized in Table 2.

**Figure 1:**
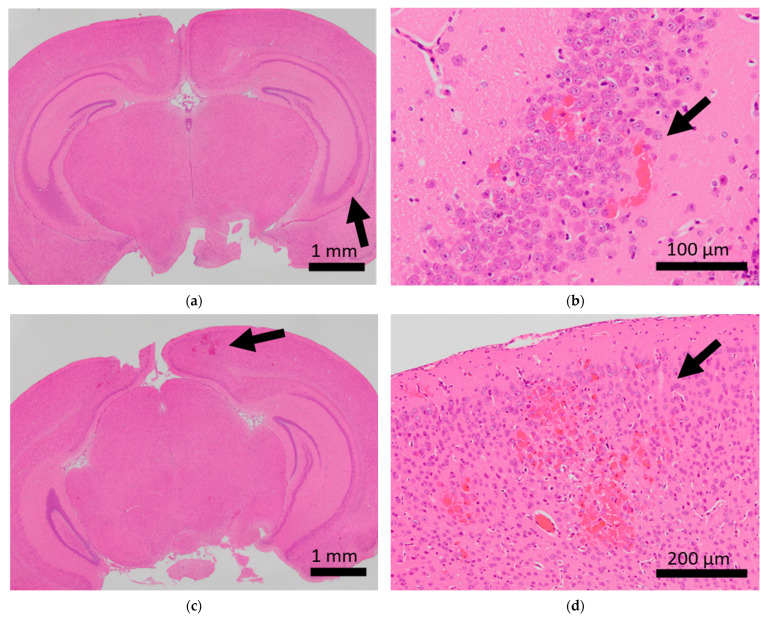
Histopathological analysis of the brains of BALB/c mice. Arrows indicate microhemorrhage in the brains of mice exposed to sCSDS. (**a**,**b**) Hippocampal microhemorrhage; (**c**,**d**) cerebral microhemorrhage.

**Figure 2:**
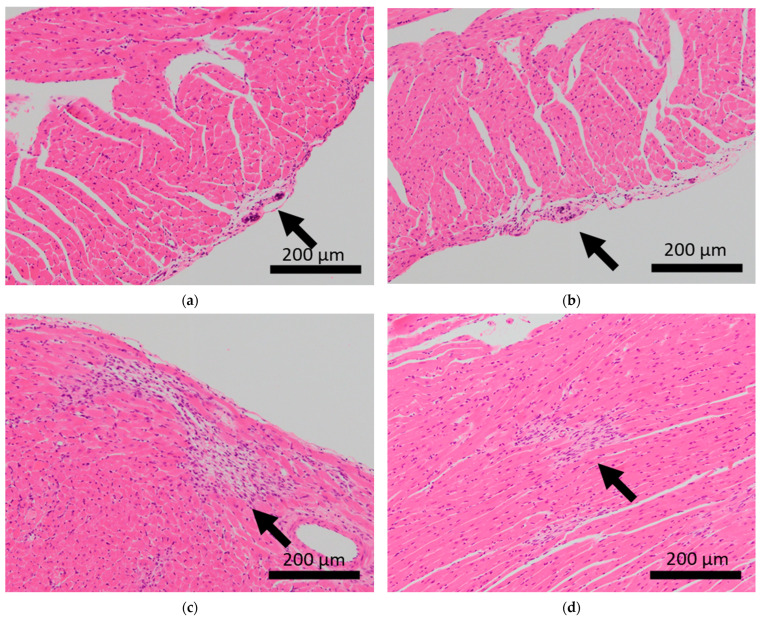
Histopathological features of the hearts of BALB/c mice. Arrows indicate calcification of the epicardium in the hearts of control mice (**a,b**), and fibrosis in mice subjected to sCSDS (**c,d**).

**Figure 3:**
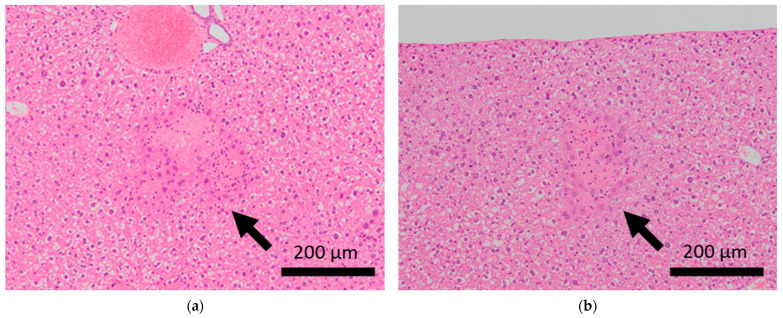

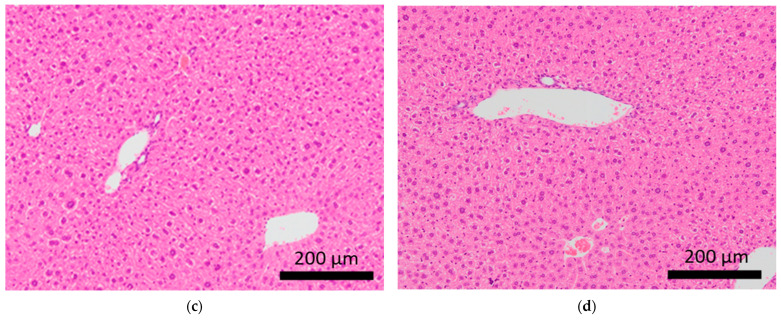
Histopathological features of the livers of BALB/c mice. Arrows indicate focal necrosis in the livers of control mice (**a,b**), whereas this was not observed in mice subjected to sCSDS (**c,d**).

**Figure 4:**
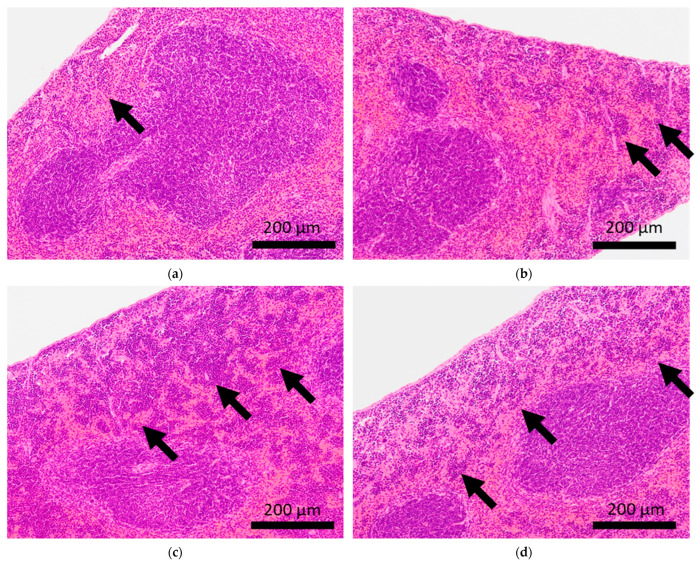
Histopathological features of the spleens of BALB/c mice. Arrows indicate extramedullary hematopoiesis in the spleens of control mice (**a,b**) and mice subjected to sCSDS (**c,d**).

Several histopathological deficits were seen in the brain, heart, liver, spleen, and lung (Table 2 and Figure 1, Figure 2, Figure 3 and Figure 4), but no remarkable damage was detected in the kidney, testis, tongue, salivary glands, bone marrow, thymus, adrenal gland, urinary bladder, adipose tissue, brown fat, skeletal muscles, stomach, intestine, or colon.

Brain microhemorrhage was observed in sCSDS mice but not in control mice (Control: 0.00% vs. sCSDS: 33.33%, *p* = 0.26). Calcification of the epicardium in the heart was observed in both control and sCSDS mice (Control: 80.00% vs. sCSDS: 44.44%, *p* = 0.30). Mild fibrosis in the heart was observed only in the sCSDS mice (Control: 0.00% vs. sCSDS: 77.78%, *p* = 0.02). Interestingly, focal necrosis in the liver was observed only in control mice, and not in sCSDS mice (Control: 100.00% vs. sCSDS: 0.00%, *p* < 0.01). In the spleen, extramedullary hematopoiesis was observed in both groups (Control: 60.00% vs. sCSDS: 100.00%; *p* = 0.11). Further, lung hemorrhage was observed in both groups (Control: 80.00% vs. sCSDS: 55.56%, *p* = 0.58).

### 2.3. Putrefactive Metabolites in Blood Plasma

The concentrations of the plasma putrefactive metabolites are listed in Table 3. Phenyl glucuronide, indoxyl glucuronide, and phenyl sulfate were not detected in blood plasma samples. Plasma *p*-cresyl glucuronide and *p*-cresyl sulfate concentrations were significantly elevated in sCSDS mice compared with control mice (*p* < 0.05).

## 3. Discussion

This study aimed to elucidate the pathological features of male BALB/c mice subjected to sCSDS using histological analysis of central and peripheral tissues and biochemical analysis of plasma putrefactive metabolites.

### 3.1. Brain

Mild brain microhemorrhage was found in two of the seven mice subjected to sCSDS, but not in control mice (Table 2 and Figure 1).

Thus, sCSDS may occasionally induce brain microhemorrhage. There is significant evidence that social defeat (SD) stress affects cerebral blood vessels. Menard et al. discovered a leaky blood–brain barrier following tight junction deficits in the nucleus accumbens of stress-susceptible mice subjected to SD and patients with depression [28]. Lehmann et al. revealed that chronic SD stress induces more severe microbleeds in the whole brain compared to our observations [29]. Thus, sCSDS may have a milder impact on the cerebral blood vessels than chronic SD stress (CSDS). However, the function of the blood–brain barrier and tight junctions in the brain of sCSDS mice should still be characterized.

### 3.2. Heart

Calcification of the epicardium was observed in both control and sCSDS mice (Table 2 and Figure 2). This pathological feature follows a previous study that revealed spontaneous cardiac calcinosis in BALB/c mice. However, the underlying mechanisms have not been elucidated [30]. Cardiac fibrosis was frequently observed in sCSDS mice (Table 2 and Table S1 and Figure 2). This also follows the results of previous studies. Costoli et al. reported that CD-1 mice subjected to CSDS showed cardiac fibrosis, and this pathogenesis might be based on the effects of adrenergic stimulation following cardioventricular remodeling [31]. Hammamieh et al. reported that C57BL/6J mice subjected to a modified CSDS paradigm showed myocardial fibrosis [32]. In our previous study, elevated hydroxyproline levels were observed in the blood plasma of sCSDS mice [17], which might indicate either increased collagen synthesis or degradation. Generally, myocardial fibrosis in humans can be induced by various stimuli, including social stress, and excessive cardiac fibrosis increases the risk of heart failure [33,34]. Therefore, the molecular mechanisms underlying the pathophysiology of cardiac fibrosis in mice subjected to SD should be further investigated.

### 3.3. Liver

Focal necrosis without inflammation was found in the livers of control mice but not in those of sCSDS mice (Table 2 and Table S1, and Figure 3). Spontaneous necrosis of the liver is frequently observed in healthy control rodents and may be induced by local hypoxia, with focal decreases in blood flow [35]. Interestingly, sCSDS may rescue this pathogenesis in the liver. Therefore, whether sCSDS affects the focal circulation of the liver must still be elucidated.

### 3.4. Spleen and Lung

Extramedullary hematopoiesis in the spleen and hemorrhage in the lungs were observed in both the control and sCSDS mice (Table 2). However, severe extramedullary hematopoiesis in the spleen was mainly induced by sCSDS (Figure 4). The body weight ratio of spleens of sCSDS mice was significantly larger than that of control mice (Control: 0.357 ± 0.012 % vs. sCSDS: 0.630 ± 0.062 %, *p* < 0.01). Consistent with this, McKim et al. reported that SD stress enlarges and increases extramedullary hematopoiesis in the spleen [36].

### 3.5. Putrefactive Metabolites in Blood Plasma

The plasma concentrations of *p*-cresyl glucuronide and *p*-cresyl sulfate were elevated by sCSDS (Table 3). These putrefactive metabolites are generated by the gut microbiota and liver from dietary tyrosine and increase the risk of CKD [37]. An in vitro study revealed that *p*-cresyl sulfate-induced renal cell damage via reactive oxygen species [38]. In this study, no significant pathological findings were observed in the kidneys of sCSDS mice. Therefore, long-term histological observations of the kidneys after sCSDS are needed. Interestingly, plasma *p*-cresyl sulfate levels in patients with CKD were associated with cardiovascular deficits [39]. Notably, Han et al. reported that *p*-cresyl sulfate-induced cardiac dysfunction in mice was caused by cardiomyocyte apoptosis and collagen accumulation [40]. Therefore, cardiovascular fibrosis in sCSDS mice may be caused by increased plasma *p*-cresyl glucuronide and *p*-cresyl sulfate.

## 4. Materials and Methods

### 4.1. Animals

This study was approved by the Animal Care and Use Committee of Ibaraki University (#20170 and #21050) and conformed to the guidelines of the Ministry of Education, Culture, Sports, Science and Technology (MEXT), Japan (Notification, No. 71).

Male BALB/cAJcl (BALB) mice (7 weeks old, experimental mice; Clea Japan, Tokyo, Japan) and Slc:ICR (ICR) mice (retired, older than five months, to defeat the BALB mice; Japan SLC, Shizuoka, Japan) were introduced into the animal facility of the College of Agriculture, Ibaraki University, under a 12 h light–dark cycle (lights on at 7:00 am). Mice were treated as described [8,9]. Before the experiments, the BALB mice were housed in individual cages (143 mm × 293 mm × 148 mm; Charles River Laboratories Japan, Kanagawa, Japan) with wood-chip bedding. BALB mice were fed a semi-purified diet (AIN-93G, Oriental Yeast, Tokyo, Japan), and ICR mice were fed a standard laboratory diet (MF) (Oriental Yeast). Feed and water were provided ad libitum to all mice. BALB mice were weighed daily to monitor the daily consumption of feed and water. Body weight was measured daily to calculate changes in body weight.

### 4.2. Experimental Design

**Figure 5:**
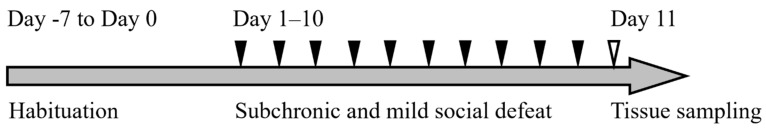
The timeline of this experiment. Mice were habituated in the animal facility (Day −7 to Day 0) and subjected to sCSDS (days 1–10). Tissue sampling was performed on day 11.

The experimental design is shown in Figure 5.

After habituation to the environment in the animal facility for one week (from day −7 to day 0), BALB mice (*n* = 14) were divided into two groups: the sCSDS (*n* = 9) and control (*n* = 5) groups. The sCSDS conditions were applied for 10 days on days 1–10 in a SD cage (220 mm × 320 mm × 135 mm; Natsume Seisakusho, Tokyo, Japan). Other BALB mice (Control: *n* = 2, sCSDS: *n* = 2) subjected to routine measurement of blood pressure and heart rate were analyzed histopathologically (Supplementary Table S1). Tissue sampling was performed on day 11.

### 4.3. sCSDS Paradigm

The sCSDS mouse model was performed as described [9,41]. The sCSDS was performed in the morning (10:00–12:00) and consisted of approximately half-scale physical contact times, compared with 5 min of physical contact for 10 days in the standard CSDS model. Briefly, physical contact was set to 5 min after the first attack bite on day 1, after which the duration was reduced in a stepwise manner by 0.5 min per day. Finally, 0.5 min of physical contact was facilitated on day 10, as described by Goto et al. [9,41]. After physical contact, the experimental (BALB) mice were moved into the compartment neighboring that of the ICR, and separated by a divider for 24 h using an SD cage.

### 4.4. Tissue Sampling

Tissue sampling was conducted after 3 h (7:00–10:00) of fasting on day 11. Animals were anesthetized by inhalation of isoflurane (5% for induction, 3% for maintenance) (Pfizer Japan Tokyo, Japan). Blood was collected in a microtube containing EDTA-2K at a final concentration of 0.13% via the abdominal vein under isoflurane anesthesia. The microtubes were subsequently centrifuged (10 min, 1200 × *g*, 4 °C), and the supernatant was collected and stored at −80 °C until analysis. Mice were euthanized via decapitation, after which tissues including brain, heart, lungs, liver, stomach, intestines, kidneys, adrenal glands, testes, white adipose tissue, bladder, abdominal vena cava, abdominal aorta, bone marrow (ribs), thymus, salivary glands, tongue, brown fat tissue, and skeletal muscle were collected and fixed with 10% neutral buffered formalin for histological analysis.

### 4.5. Histopathological Analysis

All animals (*n* = 14) were used for histopathological analysis. Formalin-fixed organs were embedded in paraffin wax, cut into four μm sections and stained with hematoxylin and eosin. Histopathological lesions were analyzed by a previous report [42] and observed under a light microscope (BX51; Olympus, Tokyo, Japan). All lesions were scored using these criteria: —, no remarkable change; +, mild; ++, moderate; +++, severe.

### 4.6. Analysis of Putrefactive Metabolites

Putrefactive metabolites—including phenyl glucuronide, indoxyl glucuronide, *p*-cresyl glucuronide, phenyl sulfate, indoxyl sulfate, and *p*-cresyl sulfate—in the plasma were analyzed using a previously described method [43].

To measure the TMAO concentration, another detection method was performed as described with some modifications [44]. Briefly, thawed plasma (50 µL) was transferred to a 1.5 mL microtube, 10 µL of internal standard solution (10 mmol/L Trimethylamine-d9 N-oxide (Cayman Chemical, Arbor, MI, USA)) was added, and the microtube was vortexed. Then, 50 µL of perchloric acid (0.4 mol/L; Fujifilm Wako, Osaka, Japan) was applied, vortexed, and incubated on ice for 30 min. After centrifugation (15,000 × *g*, 15 min, 4 °C), the supernatant was filtered using a 0.2 µm membrane filter (mdi Syringe Filter, Advanced Microdevices PVT, Cantt, India) and transferred to a vial. It was then used as a sample for TMAO detection analyses.

Plasma TMAO concentration was measured using an ultra-performance liquid chromatography device equipped with a binary solvent manager, autosampler, column heater, and tandem mass spectrometry (LC–MS/MS; Acquity TQD System, Waters, Milford, MA, USA). Chromatographic separation was conducted using an Inertsil HILIC 2.1f’150 mm column (particle size 3.0 µm; GL Sciences, Tokyo, Japan). The LC–MS/MS conditions were: solvent A, ultrapure water (Fujifilm Wako); solvent B, acetonitrile (Fujifilm Wako); column temperature, 40 °C; flow rate, 0.2 mL/min; injection volume, 5 µL; gradient program, 0–3 min: %B = 1, 3–4 min: %B = 1–60 gradient, 4–6 min: %B = 60, 6–8 min: %B = 60–100 gradient, and 8–12 min: %B = 100. MS spectra were obtained in the electrospray ionization (ESI) positive ion mode. The positive ion mode was set as follows: ion source temperature, 120 °C; capillary desolvation temperature, 350 °C; capillary voltage, 3.0 kV. The capillary desolvation and cone gas flows were set to 600 and 50 L/h, respectively. A multiple reaction monitoring transition of m/z 76.0 > 59.2 (TMAO) and 85.0 > 68.3 (TMA-d9-O) was used.

### 4.7. Statistical Analysis

Body weight gain, total feed, water intake, and concentrations of putrefactive metabolites were statistically analyzed using Student’s *t-*test. Histopathological data were analyzed using Fischer’s exact test. Differences between the means were significant if *p* < 0.05, with a tendency to be significant if 0.05 < *p* < 0.1. Data are shown as mean ± SEM and were analyzed using Excel Toukei (SSRI, Tokyo, Japan).

## 5. Conclusions

In conclusion, in mice, systemic abnormalities are induced by sCSDS, resulting in the elevation of putrefactive metabolites in the blood. Histopathological abnormalities were observed after just 11 days of stress induction; therefore, relatively short-term sCSDS might induce functional and structural abnormalities. Putrefactive metabolites are toxic materials produced by the gut microbiota, and might be associated with these observed abnormalities. In this study, we identified several pathological features of sCSDS in mice. However, this study has limitations. We used only male mice because the standard paradigm could not produce a sCSDS model in female mice. Thus, the pathophysiology of female defeat models should be investigated further. Moreover, pathological and biochemical analyses were performed immediately after sCSDS. It would undoubtedly be fruitful to observe the long-term pathological changes after sCSDS.

## Figures and Tables

**Table 1:** Feed intake, water intake, and body weight gain during the sCSDS period (Day 0–11) in BALB/c male mice (Control mice; *n* = 4, sCSDS mice; *n* = 7).

	Control	sCSDS	*p*-Value
Total feed intake (g)	31.36 ± 0.61	32.37 ± 0.85	0.36
Total water intake (g)	30.55 ± 0.76	31.85 ± 1.94	0.55
Body weight gain (g)	0.74 ± 0.33	−0.10 ± 0.66	0.29

**Table 2:** Summary of the pathological findings of the tissues in sCSDS and control mice.

	Brain	Heart	Liver	Spleen	Lung
Control 1	—	Calcification of the epicardium (++)	Focal necrosis (++)	Extramedullary hematopoiesis (+)	Hemorrhage (+)
Control 2	—	Calcification of the epicardium (+)	Focal necrosis (++)	—	Hemorrhage (+)
Control 3	—	Calcification of the epicardium (+)	Focal necrosis (++)	Extramedullary hematopoiesis (+)	Hemorrhage (+)
Control 4	—	Calcification of the epicardium (++)	Focal necrosis (+)	Extramedullary hematopoiesis (+)	Hemorrhage (+)
Control 5	—	—	Focal necrosis (+)	—	—
sCSDS 1	—	Calcification of the epicardium (+)	—	Extramedullary hematopoiesis (+++)	—
sCSDS 2	—	Fibrosis (+), Calcification of the epicardium (+)	—	Extramedullary hematopoiesis (+++)	—
sCSDS 3	—	Calcification of the epicardium (+)	—	Extramedullary hematopoiesis (++)	Hemorrhage (+)
sCSDS 4	Microhemorrhage (+)	Fibrosis (+)	—	Extramedullary hematopoiesis (+)	Pulmonary edema, Hemorrhage (+)
sCSDS 5	Microhemorrhage (+)	Fibrosis (++)	—	Extramedullary hematopoiesis (+++)	Hemorrhage (+)
sCSDS 6	—	Fibrosis (+)	—	Extramedullary hematopoiesis (+)	—
sCSDS 7	—	Fibrosis (+), Calcification of the epicardium (+)	—	Extramedullary hematopoiesis (+)	Hemorrhage (+)
sCSDS 8	Microhemorrhage (+)	Fibrosis (+)	—	Extramedullary hematopoiesis (+)	Edema (+)
sCSDS 9		Fibrosis (+)	—	Extramedullary hematopoiesis (+)	Hemorrhage (+)

—: no remarkable change; +: mild; ++: moderate; +++: severe.

**Table 3:** Effects of sCSDS on the putrefactive metabolites in blood plasma of BALB/c male mice (μmol/L plasma) (Control mice; *n* = 4, sCSDS mice; *n* = 7).

	Control	sCSDS	*p*-Value
TMAO	1.46 ± 0.34	1.00 ± 0.20	0.30
*p*-Cresyl glucuronide	0.00 ± 0.00	0.64 ± 0.21	0.02
Indoxyl sulfate	5.34 ± 0.64	3.75 ± 0.64	0.12
*p*-Cresyl sulfate	0.00 ± 0.00	3.60 ± 1.15	0.02

## Data Availability

Not applicable.

## Supplementary Materials

The following supporting information can be downloaded at: https://www.mdpi.com/article/10.3390/ijms24021237/s1.
